# Expression of human miR-200b-3p and -200c-3p in cytomegalovirus-infected tissues

**DOI:** 10.1042/BSR20180961

**Published:** 2018-12-07

**Authors:** Kyoung Hwa Lee, Beom Jin Lim, Victor H. Ferreira, Seo Yeon Min, Yeon-Mi Hong, Jeong-Hyeon Jo, Sang Hoon Han

**Affiliations:** 1 Division of Infectious Disease, Department of Internal Medicine, Yonsei University College of Medicine, Seoul, Republic of Korea; 2 Department of Pathology, Yonsei University College of Medicine, Seoul, Republic of Korea; 3 Multi-Organ Transplant Program, University Health Network, Toronto, Ontario, Canada

**Keywords:** cytomegalovirus, immediate early protein 2, microRNA, tissues

## Abstract

Human cytomegalovirus (HCMV) infection can cause inflammatory tissue-invasive end-organ diseases upon lytic replication. In humans, mature miR-200b-3p and -200c-3p suppress the synthesis of HCMV immediate early 2 (IE2) protein by binding to the 3′-UTR of the mRNA encoded by the unique long (UL) 122-123 region in human foreskin fibroblasts and pre-transplant peripheral blood mononuclear cells stimulated with HCMV. The present study aimed to quantitate the expression of *Homo sapiens* (hsa)-miR-200b-3p and 200c-3p in HCMV-infected tissues. We collected 240 HCMV-infected and 154 HCMV-non-infected, formalin-fixed, paraffin-embedded tissue samples of the gastrointestinal (GI) tract and bronchi/lungs. MiRNAs, HCMV, and glyceraldehyde-3-phosphate dehydrogenase (GAPDH) were quantitated by quantitative reverse transcription-PCR (qRT-PCR) and quantitative PCR (qPCR) on the basis of standard curves generated using miRNA mimics, the HCMV strain from National Institute for Biological Standards and Control (NIBSC) 09/162, and GAPDH control. To avoid the effect of cell counts on the qRT-PCR and qPCR results, the data were normalized to GAPDH levels. HCMV-infected tissues had significantly lower levels of 200b-3p/GAPDH (3.03 ± 1.50 compared with 3.98 ± 1.08 log_10_ copies/μl, *P*<0.001) and 200c-3p/GAPDH (4.67 ± 1.84 compared with 6.35 ± 1.47 log_10_ copies/μl, *P*<0.001) than normal tissues. The values for 200b-3p/GAPDH (*r* = −0.51, *P*<0.001) and 200c-3p/GAPDH (*r* = −0.54, *P*<0.001) were significantly inversely correlated with HCMV load. Low tissue levels of 200b-3p and 200c-3p in humans are associated with cytopathic inflammation due to HCMV infection.

## Introduction

Human cytomegalovirus (HCMV) replication can cause serious harm in solid organ transplantation (SOT) and hematopoietic stem cell transplantation (HSCT) recipients [1–3]. In particular, post-transplant HCMV viremia has various indirect immunomodulatory effects, such as chronic allograft dysfunction and failure, in SOT recipients [1,2]. HCMV reactivation has been associated with increased mortality and prolonged treatment of mechanical ventilation in critically ill intensive care unit patients without prior objective immunocompromised conditions [4–7]. This worsening of outcomes by HCMV infection has led to the implementation of regular HCMV monitoring as well as well-organized and patient-tailored preventive strategies with risk stratification in routine clinical practice of SOT and HSCT recipients [1–3]. In addition, clinical trials have been conducted for evaluating the effects of HCMV prevention on outcomes in non-immunocompromised critically ill patients with acute respiratory distress syndrome [8].

Numerous basic and clinical studies fully support the need for continuous suppression and elimination of HCMV replication, a need that is currently unmet [9]. *In vitro* experiments on cultured cells have shown that epigenetic regulation through chromatin structural changes in the major immediate-early promoter (MIEP) and miRNA originating from the human host and/or HCMV play important roles in balancing latency and cytopathic replication [10–14]. Therefore, complete blockage of the production of immediate-early (IE) protein 2 (pp86), which is essential for the lytic virion structure and is encoded by the HCMV UL122-123 region, could be an effective approach to maintaining latency [12–15].

Seed sequences of human (*Homo sapiens* (Hsa)) miRNAs bind to the 3′-UTR of certain mRNAs and generally inhibit protein synthesis [16]. *In vitro* studies revealed that hsa-mature miRNA (miR)-200b-3p, -200c-3p, and -429, belonging to the miRNA-200 family, bind the 3′-UTR of HCMV UL122-123, in accordance with *in silico* predictions, and decrease IE2 synthesis in HCMV-infected fibroblasts [12,17]. Moreover, higher levels of hsp-miR-200b-3p and -200c-3p in pre-transplant blood with *in vitro* HCMV stimulation were associated with lower post-transplant HCMV replication rates in SOT recipients [17]. These results suggest that hsa-miR-200b-3p, -200c-3p, and -429 might serve as biomarkers to predict HCMV infection and/or disease and as therapeutic targets to control HCMV replication. The present study aimed to confirm the association between the levels of hsa-miR-200b-3p or -200c-3p or -429 and HCMV load in a clinical setting, with a focus on tissue-invasive disease, using formalin-fixed paraffin-embedded (FFPE) tissues.

## Methods

### FFPE tissue collection

We collected 240 whole-block HCMV-infected FFPE tissues between January 2007 and August 2016 in Shinchon and Gangnam Severance Hospital (Seoul, Korea) on the basis of information retrospectively extracted from electronic medical records. HCMV-infected tissues were strictly defined as those distinctly positive in immunohistochemical staining (IHS) using mouse anti-HCMV monoclonal antibody (mAb) (Clone DDG9 and CCH2; Agilent Dako Technologies, Seoul, South Korea) along with cytopathic histopathological findings of inflammation with/without necrosis as well as ulcerative/erosive lesions. After the exhaustive review of pathologic reports, we excluded FFPE tissues with following features: (i) suspected viral infection as indicated by the presence of giant cells with macronuclei/smudged chromatin/inclusion bodies, but IHS for HCMV was negative or not performed; (ii) co-infections with other organisms; (iii) patient was ≤18 years of age; and (iv) pathologic findings such as malignancies and acute/chronic inflammation caused by factors other than HCMV. As controls, 154 blocks of HCMV-non-infected age- and sex-matched FFPE tissues were obtained, between 2011 and 2016, from the resection margin of solid cancers and without any abnormal histological findings were included. The present study was approved by the institutional review board with waiver of written informed consent.

### DNA and RNA preparation

Total RNA was isolated from 20-μm-thick FFPE tissue sections using a miRNeasy® FFPE Kit per the manufacturer’s instructions (Qiagen, Seoul, Korea). Genomic DNA (gDNA) was extracted with a QIAamp^®^ DNA FFPE Tissue kit per the manufacturer’s instructions (Qiagen). All RNA and gDNA samples had A_260_/A_280_ values of 1.8–2.0 as measured in a NanoDrop™ 2000 spectrophotometer (Thermo Fisher Scientific, Seoul, Korea). All samples were stored at −80°C until use.

### Quantitative real-time PCR and reverse transcription PCR

HCMV was quantitated by quantitative PCR (qPCR) using the National Institute for Biological Standards and Control (NIBSC) code 09/162 (NIBSC, Hertfordshire, U.K.) to obtain standard curves according to the World Health Organization (WHO) international standard guidelines for HCMV nucleic acid amplification techniques [18,19]. One vial of NIBSC code 09/162 was reconstituted in 1 ml, yielding 5 × 10^6^ international unit (IU)/ml [18,19]. The primers and probe for amplification of HCMV UL83 region were: forward, 5′-GCAGCCACGGGATCGTACT-3′; reverse, 5′-GGCTTTTACCTCACACGAGCATT-3′; probe, 5′-6(FAM)-CGCGAGACCGTGGAACTGCG-(TAMRA)-3′ (Applied Biosystems/Thermo Fisher Scientific, Foster City, CA, U.S.A.) [20,21]. qPCRs were conducted using 50 ng/μl of gDNA, and for the standard curve, 5 × 10^1^ to 5 × 10^6^ IU/ml, and TaqMan™ universal PCR master mixture under the following conditions: 2 min at 50°C, 10 min at 95°C, 45 cycles of 15 s at 95°C, and 1 min at 60°C [20,21].

Quantitative reverse transcription-PCR (qRT-PCR) of hsa-miR-200b-3p, -200c-3p, and -429 was performed with stem-loop primers and probes from TaqMan™ Small RNA Assays (Assay IDs: 002251, 002300, and 001024, respectively) as well as TaqMan™ universal PCR master mixture (Applied Biosystems/Thermo Fisher Scientific). Using 6 ng/μl of total RNA, RT was performed in a C1000 Touch™ thermal cycler (Bio-Rad, Seoul, Korea) under the following conditions: 30 min at 16°C, 30 min at 42°C, and 5 min at 85°C. qRT-PCRs were run using 1.33 μl of cDNA, with the following thermal cycles: 10 min at 95°C, 45 cycles of 15 s at 95°C, and 60 s at 60°C. Standard curves for quantitation were generated using mirVana™ miRNA mimics [22] at 10^1^–10^9^ copies/μl.

To exclude the effects of severity of necrosis on HCMV quantity [14,23], we measured the expression of the housekeeping protein glyceraldehyde-3-phosphate dehydrogenase (GAPDH) in 100 ng/μl of gDNA through qPCR using TaqMan™ human GAPDH endogenous control (6FAM™/MGB probe, non-primer limited) according to the manufacturer’s instructions (Applied Biosystems/Thermo Fisher Scientific). qPCRs were run in a LightCycler^®^ 480 platform (Roche Diagnostics, Seoul, Korea). Each sample was tested in triplicate. Reliability analysis using inter-class correlation of *C*_t_ values for each qRT-PCR or qPCR showed nearly perfect reproducibility as 0.996 for hsa-miR-200b-3p, 0.994 for -200c-3p, 0.987 for HCMV, and 0.959 for GAPDH.

The quantity of miRNAs or GAPDH was expressed as log_10_ copies/μl of input RNA or log_10_ copies/μl of input DNA. The HCMV load was expressed as log_10_ IU/μl of input DNA. The limit of detection of all qRT-PCRs was defined as 2 log_10_ copies/μl. All data under this threshold were considered as undetectable for categorical and zero for continuous variable analysis.

## Statistical analyses

Data were expressed as mean ± S.D. or median (interquartile range) or number (percent). Statistical analyses were performed using SPSS V23.0 (SPSS, Chicago, IL, U.S.A.) and GraphPad Prism V6 (GraphPad Software, La Jolla, CA). Differences in categorical and continuous variables amongst two groups were analyzed by chi-square and independent *t* test or Mann–Whitney U test, respectively. We used one-way ANOVA test and post-hoc Bonferroni’s multiple comparison tests to compare three groups. Correlations between two continuous variables were expressed as Pearson’s *r* value and 95% confidence interval (CI). To adjust the type I error by multiple independent comparisons, we considered the significant two-tailed *P*-value as less than 0.002 (0.05/25 of independent comparisons in the present study).

## Results

### Clinical features of patients and FFPE tissues

Table 1 summarizes the clinical characteristics of patients and FFPE tissues. All tissues originated from HCMV seropositive subjects. Mean age (*P*=0.188) and sex (*P*=0.872) were not different between HCMV-infected and non-infected tissues. The majority of tissues originated from the gastrointestinal (GI) tract, including the esophagus, stomach, colon, and rectum, and there was no significant difference in organ distribution between the two groups (*P*=0.918).

**Table 1:** Characteristics of HCMV-infected and -non-infected, normal FFPE tissues

Characteristics	Total (*n*=394)	HCMV-infected (*n*=240)	Normal (*n*=154)	*P-*value
Age, years	57.7 ± 17.2	56.8 ± 16.9	59.2 ± 17.7	0.188^1^
Sex, male (%)	256 (65.0)	154 (64.2)	102 (66.2)	0.872^2^
Tissue type				0.918^2,3^
GI tract	370 (93.9)	235 (97.9)	135 (87.7)	
Upper	122 (31.0)	67 (27.9)	56 (36.4)	
Esophagus	58 (14.7)	31 (12.9)	27 (17.5)	
Stomach	64 (16.2)	36 (15.0)	29 (18.9)	
Lower	248 (62.9)	168 (65.8)	79 (51.3)	
Small intestine	13 (3.3)	13 (5.4)	0 (0.0)	
Large intestine	235 (59.6)	155 (64.6)	79 (51.3)	
Colon	164 (41.6)	104 (43.3)	54 (35.1)	
Rectum	71 (18.0)	51 (21.3)	25 (16.2)	
Lung and bronchus	24 (6.1)	5 (2.1)	19 (12.3)	

Data are expressed as mean ± S.D. or number (percent). ^1^Independent *t* test. ^2^Chi-square test. ^3^Comparison between three variables of upper GI tract, lower GI tract, and lung/bronchus.

### miRNA and HCMV levels in HCMV-infected and normal FFPE tissues

**Figure 1:**
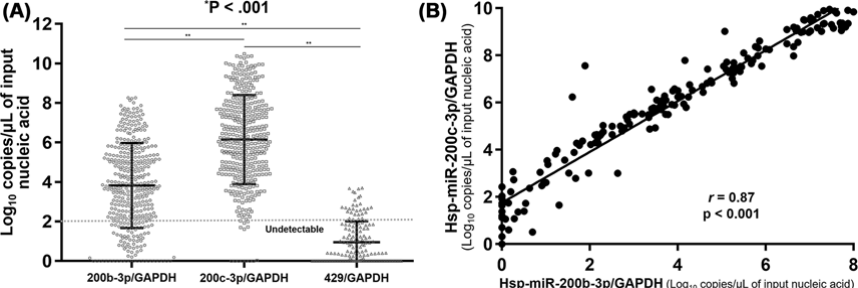
Differences and correlation between levels of hsa-miR-200b-3p, -200c-3p, and -429 in 394 analyzed tissues samples (**A**) Differences in hsa-miR-200b-3p, -200c-3p, and -429 levels after normalization to GAPDH. Bars represent means and S.D. Dotted line indicates limit of detection (100 copies/μl of input nucleic acid). *P-value between three miRs by ANOVA test, **P<0.001 between two miRs by Bonferroni’s multiple comparisons tests. (**B**) Correlation between 200b-3p and 200c-3p after normalization to GAPDH. The levels of hsa-miR-200b-3p/GAPDH and -200c-3p/GAPDH are strongly correlated, with *r* = 0.87 (95% CI, 0.86–0.87) and *P*<0.001. ‘Nucleic acid’ stands for DNA for GAPDH and RNA for miRNAs. Each dot corresponds to the miRNA/GAPDH level expressed as log_10_ copies/μl of input nucleic acid in all figures.

The levels of hsa-miR-200c-3p (7.19 ± 1.71 log_10_ copies/μl) in all 394 tissues were significantly (*P*<0.001) higher than those of 200b-3p (4.76 ± 1.47 log_10_ copies/μl). The 200c-3p/GAPDH had significantly higher values compared with 200b-3p/GAPDH (6.15 ± 2.26 compared with 3.83 ± 2.15 log_10_ copies/μl) with a 211-fold difference. The levels of miR-429/GAPDH (0.95 ± 0.85 log_10_ copies/ μl) was lowest amongst three miRs (*P*<0.001) (Figure 1A). Because few samples (34 of 394, 8.6%) had expression levels above limit of detection for miR-429, we performed additional analyses with 200b-3p and -200c-3p. The expression of 200b-3p and 200c-3p showed a strong positive correlation (*r* = 0.85, 95% CI, 0.82–0.88, *P*<0.001), even when the level of each miRNA was normalized to that of GAPDH mRNA in all 394 tissues (*r* = 0.87, 95% CI, 0.86–0.87, *P*<0.001) (Figure 1B). The HCMV load in the 240 HCMV-infected FFPE tissues was 6.15 ± 1.19 (range: 4.11–9.69) log_10_ IU/ml, whereas that in all normal tissues was less than 0.7 log_10_ IU/ml of the lower limit of quantitation.

**Figure 2:**
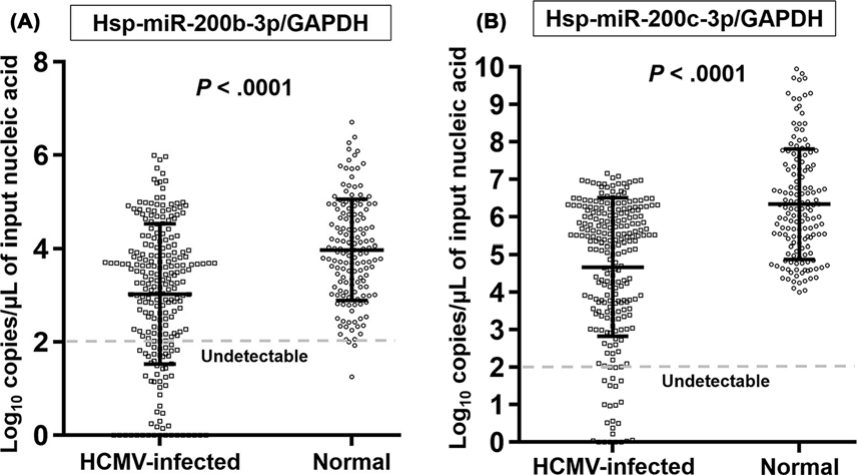
Difference of hsa-miR-200b-3p and -200c-3p levels between HCMV-infected (*n*=240) and non-infected (*n*=154) FFPE tissues **(A), (B)** Bars indicate means and S.D. Dotted lines represent limit of detection (100 copies/μl of input nucleic acid). ‘Nucleic acid’ stands for DNA for GAPDH and RNA for miRNAs. Each dot corresponds to the miRNA/GAPDH level expressed log_10_ copies/μl of input nucleic acid in all figures.

HCMV-infected tissues had significantly lower levels of both hsa-miR-200b-3p (4.26 ± 1.44 compared with 5.55 ± 1.13 log_10_ copies/μl, *P*<0.001) and 200c-3p (6.50 ± 1.60 compared with 8.26 ± 1.27 log_10_ copies/μl, *P*<0.001) than normal tissues, with 19.5- and 57.5-fold differences, respectively. After normalization to GAPDH, fold-differences in both 200b-3p and 200c-3p were decreased by 8.9 and 47.9, respectively (200b-3p/GAPDH: 3.03 ± 1.50 compared with 3.98 ± 1.08 log_10_ copies/μl, *P*<0.001 and 200c-3p; 4.67 ± 1.84 compared with 6.35 ± 1.47 log_10_ copies/μL, *P*<0.001) (Figure 2A,B). The number of samples with levels below the detection limit for hsa-miR-200b-3p (9.2 compared with 0.0%, *P*<0.001) and 200b-3p/GAPDH (23.3 compared with 1.3%, *P*<0.001) as well as 200c-3p (3.3 compared with 0.0%, *P*=0.025) and 200c-3p/GAPDH (10.0 compared with 0.0%, *P*<0.001) was significantly higher in the HCMV-infected tissues.

### Hsa-miR-200b-3p and -200c-3p levels according to tissue type

**Figure 3:**
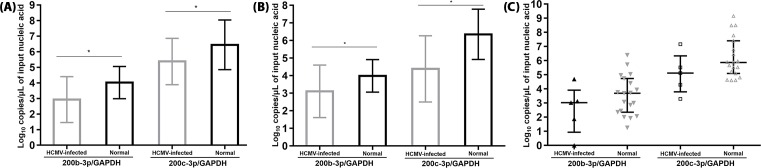
Difference in hsa-miR-200b-3p and -200c-3p levels according to tissue type (**A**) Upper-GI tract samples originating from esophagus and stomach, including 67 HCMV-infected and 56 non-infected FFPE tissues. The percentage of samples with undetectable level of hsa-miR- 200b-3p/GAPDH (21.7 compared with 0.0%) was significantly higher in HCMV-infected than non-infected upper-GI tract tissues (*P*<0.001). However, the percentage of undetectable 200c-3p/GAPDH (4.3 compared with 0.0%) was similar between the two groups (*P*=0.121). (**B**) Lower-GI tract samples from duodenum, cecum, terminal ileum, colon, and rectum, including 168 HCMV-infected and 79 normal FFPE tissues. The percentage of samples with undetectable level of hsa-miR-200b-3p/GAPDH (23.0 compared with 0.0%) was significantly higher in HCMV-infected than non-infected lower-GI-tract tissues (*P*<0.001). For 200c-3p, values normalized to GAPDH had a significantly high undetectable percentage (12.1 compared with 0.0%, *P*<0.001). (**C**) Lung samples, including 5 HCMV-infected and 19 normal FFPE tissues. In lung, the 200b-3p/GAPDH and 200c-3p/GAPDH levels did not show significant difference between two groups (*P*=0.297 and 0.120, respectively). *P<0.001. *P*-values for GI tract and lung samples were obtained through independent *t* test and Mann–Whitney U test, respectively. In (A,B), height of boxes and upper/lower lines indicate mean and S.D. In (C), bars represent median and 25 or 75 percentile values. Each dot corresponds to the miRNA/GAPDH levels expressed as log_10_ copies/μl of input nucleic acid in all figures.

HCMV-infected tissues from the upper GI tract (*n*=70) had significantly lower levels of hsa-miR-200b-3p/GAPDH (2.93 ± 1.47 compared with 4.02 ± 1.03 log_10_ copies/μl, *P*<0.001) than normal upper GI tissues (*n*=56). The levels of 200c-3p/GAPDH (5.38 ± 1.49 compared with 6.44 ± 1.59 log_10_ copies/μl, *P*<0.001) were significantly lower in HCMV-infected than in normal upper GI tissues (Figure 3A). 200b-3p/GAPDH (3.11 ± 1.49 compared with 4.00 ± 0.93 log_10_ copies/μl, *P*<0.001) and 200c-3p/GAPDH (4.39 ± 1.88 compared with 6.35 ± 1.43 log_10_ copies/μl, *P*<0.001) were significantly lower in HCMV-infected lower GI tissues (*n*=165) than in normal lower GI tissues (*n*=79) (Figure 3B). Amongst the lung tissue samples, 200b-3p/GAPDH (*P*=0.297) and 200c-3p/GAPDH (*P*=0.120), levels were not different between HCMV-infected (*n*=5) and normal (*n*=19) tissues (Figure 3C).

### Association of hsa-miR-200b-3p and -200c-3p expression with HCMV levels

**Figure 4:**
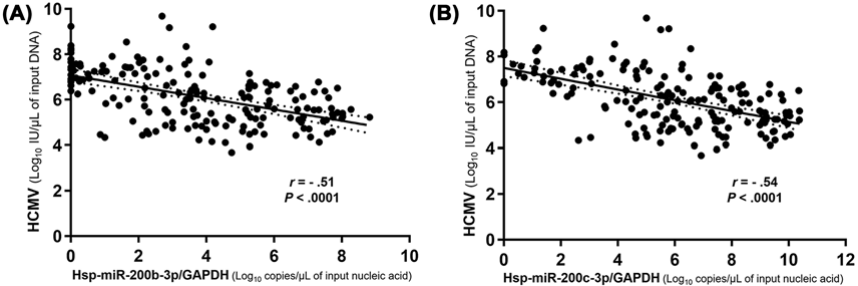
Correlation between HCMV and hsa-miR-200b-3p or -200c-3p levels in HCMV-infected FFPE tissues Straight and dotted lines represent each equation according to *r* coefficient and 95% CI values, respectively. Each dot corresponds to the miRNA/GAPDH or HCMV level expressed as log_10_ copies/μl of input nucleic acid or DNA in all figures. ‘Nucleic acid’ stands for DNA for GAPDH and RNA for miRNAs.

Both hsa-miR-200b-3p and -200c-3p were negatively correlated with HCMV levels (200b-3p; *r* = −0.69, 95% CI = −0.76 to −0.60, *P*<0.001, 200c-3p; *r* = −0.68, 95% CI = −0.74 to −0.58, *P*<0.001) in HCMV-infected tissues. The correlation coefficients after normalization to GAPDH maintained the statistical significance (200b-3p/GAPDH; *r* = −0.51, 95% CI = −0.61 to −0.39, *P*<0.001, 200c-3p; *r* = −0.54, 95% CI = −0.64 to −0.042, *P*<0.001) (Figure 4A,B).

## Discussion

Our study revealed that the expression of hsa-miR-200b-3p and -200c-3p was decreased in HCMV-infected tissues. This finding is in-line with those in two previous studies that evaluated the levels of two miRNAs in pre-transplant peripheral blood mononuclear cells (PBMCs) from SOT recipients with *in vitro* HCMV stimulation, and performed co-transfection assays using a laboratory HCMV strain and two miRNA mimics in fibroblasts [12,17]. This is the first study using HCMV-infected FFPE tissues, which are more representative of the clinical situation because these tissues definitely indicate HCMV end-organ disease.

The suppressive role of hsa-miR-200b-3p and -200c-3p on HCMV lytic replication was supported by two stepwise objective bases: (i) accurate prediction through *in silico* analysis of miRNAs binding to the 3′-UTR in the HCMV UL122-123 rather than high-throughput screening methods, such as oligonucleotide microarrays, in which expression profiles could be biased due to sample characteristics, platforms, or statistical analyses [24,25], (ii) verification of the binding of identical seed sequences in hsa-miR-200b-3p and -200c-3p to the HCMV UL122-123 by luciferase reporter assay using wild-type and mutant recombinant vectors, the results of which precisely corresponded with *in silico* prediction [12,17].

The results of the different experiments in the present study were consistent, allowing us to derive a reasonable conclusion. In spite of the relatively small differences in hsa-miR-200b-3p or -200c-3p levels between HCMV-infected and non-infected upper GI tissues, the levels of both miRNAs were significantly decreased in HCMV-infected upper/lower GI tissues. These findings were consistent between the quantitative and categorical analyses. We identified a strong negative correlation between the two miRNAs and HCMV levels; however, we were not able to establish that this correlation is directly caused by a reduction in IE2 production. This inability could be attributed to the fact that the HCMV-infected tissues do not have measurable levels of IE2 owing to the presence of large numbers of complete virions. In fact, we could not detect the mRNA encoding IE2 by qRT-PCR (data not shown). Finally, normalization of the expression data to that of GAPDH did not significantly alter the findings [14,23]. Our findings suggest that hsa-miR-200b-3p and -200c-3p could play relevant roles in regulating the initiation of HCMV active replication and/or latent-to-lytic switch by blocking the synthesis of major IE2 protein. This IE2 suppression could result in a serial reduction in early and late proteins, including HCMV DNA polymerase or glycoproteins [26]. Finally, this process could inhibit the extracellular budding of complete HCMV virion with envelope. However, the cross-reaction between these hsa-miRNAs and miRNAs encoded by HCMV, as well as the effect of MIEP, a strong transcription factor for major IE proteins, should be explored [27–30].

The major limitation of the present study would be the quality of RNA extracted from the FFPE tissues. MiRNAs can be degraded in FFPE tissues, and this damage would affect qRT-PCR results [31,32]. We did not check RNA integrity number by capillary electrophoresis. However, in addition to good A_260_/A_280_ values, the following features were considered while concluding that our RNA samples were of sufficient quality to yield satisfactory results: (i) triplicate qRT-PCRs and qPCRs for all samples showed nearly perfect inter-class correlation of *C*_t_ values; (ii) very strong correlation between the levels of hsa-miR-200b-3p and -200c-3p, with *r* = 0.85; and (iii) 200c-3p levels were higher than 200b-3p levels, similar to the findings in human PBMCs [17]. Most tissue samples were of the GI tract and a small number of the lungs, because clinical specimens of HCMV-infected tissues such as liver, lung, and central nervous system, are hard to obtain. Despite these limitations, this first evaluation of hsa-miR-200b-3p and -200c-3p in end-organ FFPE tissues gives insights in HCMV infection in association with these miRNAs in the real clinical setting.

In conclusion, the levels of hsa-miR-200b-3p and -200c-3p are reduced in HCMV-infected tissues. These miRNAs likely play a biologically relevant role in the control of HCMV after transplantation.

## Clinical perspectives

- HCMV infection can result in cytopathic tissue-invasive inflammatory diseases. In addition, active replication of HCMV is closely associated with mortality and several morbidities such as graft rejection/dysfunction/failure, graft-versus-host disease, increase in other opportunistic infections in recipients of SOT and HSCT.
- hsa-miR-200b-3p and -200c-3p suppressed the synthesis of IE2 by binding the 3′-UTR of mRNA encoded by the HCMV UL122-123 region in human foreskin fibroblasts (*in vitro*) and in pre-transplant PBMCs stimulated by HCMV (in SOT recipients).
- MiR-200b-3p and -200c-3p levels were lower in HCMV-infected tissues than in normal tissues. The levels of miR-200b-3p and -200c-3p were inversely correlated with HCMV load. These miRNAs likely play a biologically relevant role in the control of cytopathic inflammation or HCMV infection after transplantation.

## Abbreviations

CI: confidence interval
FFPE: formalin-fixed paraffin-embedded
GAPDH: glyceraldehyde-3-phosphate dehydrogenase
gDNA: genomic DNA
GI: gastrointestinal
HCMV: human cytomegalovirus
hsa: *Homo sapiens*
HSCT: hematopoietic stem cell transplantation
IE: immediate early protein
IHS: immunohistochemical staining
miR: mature microRNA
NIBSC: National Institute for Biological Standards and Control
PBMC: peripheral blood mononuclear cell
qPCR: quantitative PCR
qRT-PCR: quantitative reverse transcription-PCR
SOT: solid organ transplantation
UL: unique long

## References

[1] Razonable R.R. and Humar A. (2013) Cytomegalovirus in solid organ transplantation. Am. J. Transplant. 13, 93–106 10.1111/ajt.12103 23465003

[2] Kotton C.N. (2013) Updated international consensus guidelines on the management of cytomegalovirus in solid-organ transplantation. Transplantation 96, 333–360 10.1097/TP.0b013e31829df29d 23896556

[3] Fuji S., Einsele H. and Kapp M. (2017) Cytomegalovirus disease in hematopoietic stem cell transplant patients: current and future therapeutic options. Curr. Opin. Infect. Dis. 30, 372–376 10.1097/QCO.0000000000000375 28505028

[4] Kalil A.C. and Florescu D.F. (2009) Prevalence and mortality associated with cytomegalovirus infection in nonimmunosuppressed patients in the intensive care unit. Crit. Care Med. 37, 2350–2358 10.1097/CCM.0b013e3181a3aa43 19531944

[5] Limaye A.P. (2008) Cytomegalovirus reactivation in critically ill immunocompetent patients. JAMA 300, 413–422 10.1001/jama.2008.697 18647984 PMC2774501

[6] Papazian L. (2016) Cytomegalovirus reactivation in ICU patients. Intensive Care Med. 42, 28–37 10.1007/s00134-015-4066-9 26424680 PMC7095171

[7] Ong D.S. (2016) Cytomegalovirus reactivation and mortality in patients with acute respiratory distress syndrome. Intensive Care Med. 42, 333–341 10.1007/s00134-015-4071-z 26415682 PMC4747999

[8] Limaye A.P. (2017) Effect of ganciclovir on IL-6 levels among cytomegalovirus-seropositive adults with critical illness: a randomized clinical trial. JAMA 318, 731–740 10.1001/jama.2017.10569 28829877 PMC5817487

[9] Griffiths P.D. (2012) Burden of disease associated with human cytomegalovirus and prospects for elimination by universal immunisation. Lancet Infect. Dis. 12, 790–798 10.1016/S1473-3099(12)70197-4 23017365

[10] Reeves M.B. (2011) Chromatin-mediated regulation of cytomegalovirus gene expression. Virus Res. 157, 134–143 10.1016/j.virusres.2010.09.019 20875471 PMC5419498

[11] Kumar A. and Herbein G. (2014) Epigenetic regulation of human cytomegalovirus latency: an update. Epigenomics 6, 533–546 10.2217/epi.14.41 25431945

[12] O’Connor C.M., Vanicek J. and Murphy E.A. (2014) Host microRNA regulation of human cytomegalovirus immediate early protein translation promotes viral latency. J. Virol. 88, 5524–5532 10.1128/JVI.00481-14 24599990 PMC4019081

[13] Moller R. (2018) miRNA-mediated targeting of human cytomegalovirus reveals biological host and viral targets of IE2. Proc. Natl. Acad. Sci. U.S.A. 115, 1069–1074 10.1073/pnas.1719036115 29339472 PMC5798380

[14] Lau B. (2016) Human cytomegalovirus miR-UL112-1 promotes the down-regulation of viral immediate early-gene expression during latency to prevent T-cell recognition of latently infected cells. J. Gen. Virol. 97, 2387–2398 10.1099/jgv.0.000546 27411311 PMC5756489

[15] Paulus C. and Nevels M. (2009) The human cytomegalovirus major immediate-early proteins as antagonists of intrinsic and innate antiviral host responses. Viruses 1, 760–779 10.3390/v1030760 21994568 PMC3185523

[16] Bartel D.P. (2004) MicroRNAs: genomics, biogenesis, mechanism, and function. Cell 116, 281–297 10.1016/S0092-8674(04)00045-5 14744438

[17] Han S.H. (2017) Human microRNA responses predict cytomegalovirus replication following solid organ transplantation. J. Infect. Dis. 215, 537–546 28003351 10.1093/infdis/jiw596

[18] Fryer J.F., Heath A.B. and Minor P.D. (2016) A collaborative study to establish the 1st WHO International Standard for human cytomegalovirus for nucleic acid amplification technology. Biologicals 44, 242–251 10.1016/j.biologicals.2016.04.005 27179913

[19] Fryer J.F., Heath A.B., Anderson R., Minor P.D. and The Collaborative Study Group (2010) Collaborative study to evaluate the proposed 1st WHO International Standard for human cytomegalovirus (HCMV) for nucleic acid amplification (NAT)-based assays. WHO ECBS Report, WHO/BS/10.2138

[20] Smithers-Sheedy H. (2017) Congenital cytomegalovirus among children with cerebral palsy. J. Pediatr. 181, 267–271 10.1016/j.jpeds.2016.10.024 27816221

[21] Griscelli F. (2001) Quantification of human cytomegalovirus DNA in bone marrow transplant recipients by real-time PCR. J. Clin. Microbiol. 39, 4362–4369 10.1128/JCM.39.12.4362-4369.2001 11724846 PMC88550

[22] Di Martino M.T. (2016) Functional analysis of microRNA in multiple myeloma. Methods Mol. Biol. 1375, 181–194 10.1007/7651_2015_250 25971914

[23] Zhang J. (2013) Allitridin inhibits human cytomegalovirus replication *in vitro*. Mol. Med. Rep. 7, 1343–1349 10.3892/mmr.2013.1328 23426791

[24] Balazsi G. and Oltvai Z.N. (2007) A pitfall in series of microarrays: the position of probes affects the cross-correlation of gene expression profiles. Methods Mol. Biol. 377, 153–162 10.1007/978-1-59745-390-5_9 17634615

[25] Jaksik R. (2015) Microarray experiments and factors which affect their reliability. Biol. Direct 10, 46 10.1186/s13062-015-0077-2 26335588 PMC4559324

[26] Landais I. and Nelson J.A. (2013) Functional genomics approaches to understand cytomegalovirus replication, latency and pathogenesis. Curr. Opin. Virol. 3, 408–415 10.1016/j.coviro.2013.06.002 23816389 PMC3748260

[27] Martinez F.P. (2014) CTCF binding to the first intron of the major immediate early (MIE) gene of human cytomegalovirus (HCMV) negatively regulates MIE gene expression and HCMV replication. J. Virol. 88, 7389–7401 10.1128/JVI.00845-14 24741094 PMC4054410

[28] Ng K.R., Li J.Y. and Gleadle J.M. (2015) Human cytomegalovirus encoded microRNAs: hitting targets. Expert Rev. Anti Infect. Ther. 13, 1469–1479 10.1586/14787210.2015.1106939 26509290

[29] Shen K. (2018) Human cytomegalovirus-encoded miR-UL112 contributes to HCMV-mediated vascular diseases by inducing vascular endothelial cell dysfunction. Virus Genes 54, 172–181 10.1007/s11262-018-1532-9 29330663

[30] Stinski M.F. and Isomura H. (2008) Role of the cytomegalovirus major immediate early enhancer in acute infection and reactivation from latency. Med. Microbiol. Immunol. 197, 223–231 10.1007/s00430-007-0069-7 18097687

[31] Evers D.L. (2011) Paraffin embedding contributes to RNA aggregation, reduced RNA yield, and low RNA quality. J. Mol. Diagn. 13, 687–694 10.1016/j.jmoldx.2011.06.007 21884819 PMC3194049

[32] Howe K. (2017) Extraction of miRNAs from Formalin-Fixed Paraffin-Embedded (FFPE) tissues. Methods Mol. Biol. 1509, 17–24 10.1007/978-1-4939-6524-3_3 27826914

